# Blind killing of both male and female *Drosophila* embryos by a natural variant of the endosymbiotic bacterium *Spiroplasma poulsonii*

**DOI:** 10.1111/cmi.13156

**Published:** 2020-01-23

**Authors:** Florent Masson, Sandra Calderon‐Copete, Fanny Schüpfer, Aurélien Vigneron, Samuel Rommelaere, Mario G. Garcia‐Arraez, Juan C. Paredes, Bruno Lemaitre

**Affiliations:** ^1^ Global Health Institute, School of Life Sciences École Polytechnique Fédérale de Lausanne (EPFL) Lausanne Switzerland; ^2^ Center for Integrative Genomics Lausanne Genomic Technologies Facility Lausanne Switzerland; ^3^ Department of Epidemiology of Microbial Diseases Yale School of Public Health New Haven Connecticut; ^4^ Present address: International Centre of Insect Physiology and Ecology (ICIPE) Kasarani Nairobi Kenya

**Keywords:** endosymbiosis, male killing, Spaid, *Spiroplasma*

## Abstract

*Spiroplasma poulsonii* is a vertically transmitted endosymbiont of *Drosophila melanogaster* that causes male‐killing, that is the death of infected male embryos during embryogenesis. Here, we report a natural variant of *S. poulsonii* that is efficiently vertically transmitted yet does not selectively kill males, but kills rather a subset of all embryos regardless of their sex, a phenotype we call ‘blind‐killing’. We show that the natural plasmid of *S. poulsonii* has an altered structure: *Spaid*, the gene coding for the male‐killing toxin, is deleted in the blind‐killing strain, confirming its function as a male‐killing factor. Then we further investigate several hypotheses that could explain the sex‐independent toxicity of this new strain on host embryos. As the second non‐male‐killing variant isolated from a male‐killing original population, this new strain raises questions on how male‐killing is maintained or lost in fly populations. As a natural knock‐out of *Spaid*, which is unachievable yet by genetic engineering approaches, this variant also represents a valuable tool for further investigations on the male‐killing mechanism.

## 1 INTRODUCTION

Insects frequently harbour symbiotic bacterial population within their tissues (Buchner, 1965; Kikuchi, 2009). Most of these endosymbionts are vertically transmitted (Szklarzewicz & Michalik, 2017), increasing the stability of their association with their hosts on evolutionary timescales. Endosymbionts can strongly affect the host physiology, including its metabolic capabilities or its ability to fight against pathogens (Douglas, 2015). Some have also evolved selfish strategies to increase their prevalence in natural population by manipulating their host reproduction. Four reproductive manipulation strategies have been associated to bacterial endosymbionts to date, including feminisation, parthenogenesis, cytoplasmic incompatibility and male‐killing (MK) (Werren, Baldo, & Clark, 2008). Although the molecular mechanisms underlying these strategies are different (Harumoto & Lemaitre, 2018; LePage et al., 2017), all have as a consequence an increase in the proportion of infected individuals in host populations.

One of the most frequently reported endosymbiont that manipulates insect reproduction is *Spiroplasma* spp. The genus *Spiroplasma* is highly diverse and comprises species that are strictly pathogenic for plants and arthropods (Gasparich, 2002), but also some vertically transmitted endosymbiotic species estimated to infect 4–7% of insects (Duron et al., 2008). *Spiroplasma poulsonii* along with *Wolbachia* is one of the two endosymbionts that can naturally infect *Drosophila melanogaster* (Mateos et al., 2006). Screenings of natural *Drosophila* populations indicate a highly variable rate of infection by *Spiroplasma*, ranging from complete absence to up to 60% in some populations (Watts, Haselkorn, Moran, & Markow, 2009).

Some strains of *S. poulsonii* cause MK in *Drosophila*, whereby all male embryos die during early embryogenesis (Montenegro, Solferini, Klaczko, & Hurst, 2005). The recently unravelled molecular mechanism of MK involves a *Spiroplasma*‐encoded toxin, Spaid, that potentially targets the male‐specific lethal complex, a part of the X‐chromosome dosage compensation system in *Drosophila* (Harumoto & Lemaitre, 2018). Spaid causes abnormal segregation and breakage of X chromatids in male embryos, leading to a massive DNA‐damage dependent apoptosis and eventually to the death of the embryo (Harumoto, Anbutsu, Lemaitre, & Fukatsu, 2016; Harumoto & Lemaitre, 2018).

*S. poulsonii* also affects adult *Drosophila* physiology by reducing its lifespan and causing a neurodegenerative phenotype in old flies (Herren & Lemaitre, 2011). The cause of these two phenotypes is not clearly unravelled yet, although the involvement of cardiolipins or a neurotoxic protein released by *Spiroplasma* have been proposed as putative causes (Herren & Lemaitre, 2011; Masson, Calderon Copete, Schüpfer, Garcia‐Arraez, & Lemaitre, 2018). Last, a remarkable protection is conferred to *Spiroplasma*‐infected flies against parasitoid wasp and nematode infections (Ballinger & Perlman, 2017; Hamilton, Peng, Boulanger, & Perlman, 2016; Xie, Butler, Sanchez, & Mateos, 2014). Competition between parasites and *Spiroplasma* for host lipids has been proposed as a mechanism underlying this protection (Paredes, Herren, Schüpfer, & Lemaitre, 2016). Another hypothesis involves *Spiroplasma* toxins belonging to the Ribosome‐Inactivating Protein (RIP) family, which accumulate in the hemolymph and target parasitic wasp and nematode ribosomes (Ballinger & Perlman, 2017; Hamilton et al., 2016). The effect of *S. poulsonii* infection on *Drosophila* physiology are thus highly relying on *Spiroplasma* secreted toxins, but the lack of genetic tools to manipulate the bacteria renders it difficult to assess precisely the role of each bacterial toxin towards each phenotype.

In this study, we describe a spontaneous mutant derived from a male‐killer *S. poulsonii*. This strain causes an alternate phenotype in *Drosophila* whereby some infected offspring is killed regardless of the sex of the embryo. We show that the mutant has a clean deletion of *Spaid*, thus presenting a unique opportunity to study the equivalent of a *Spaid* full knock‐out, which is technically not achievable yet by genetic engineering.

## 2 RESULTS

### 2.1 Discovery and phenotypic characterisation of a *S. poulsonii* spontaneous mutant

**Figure 1:**
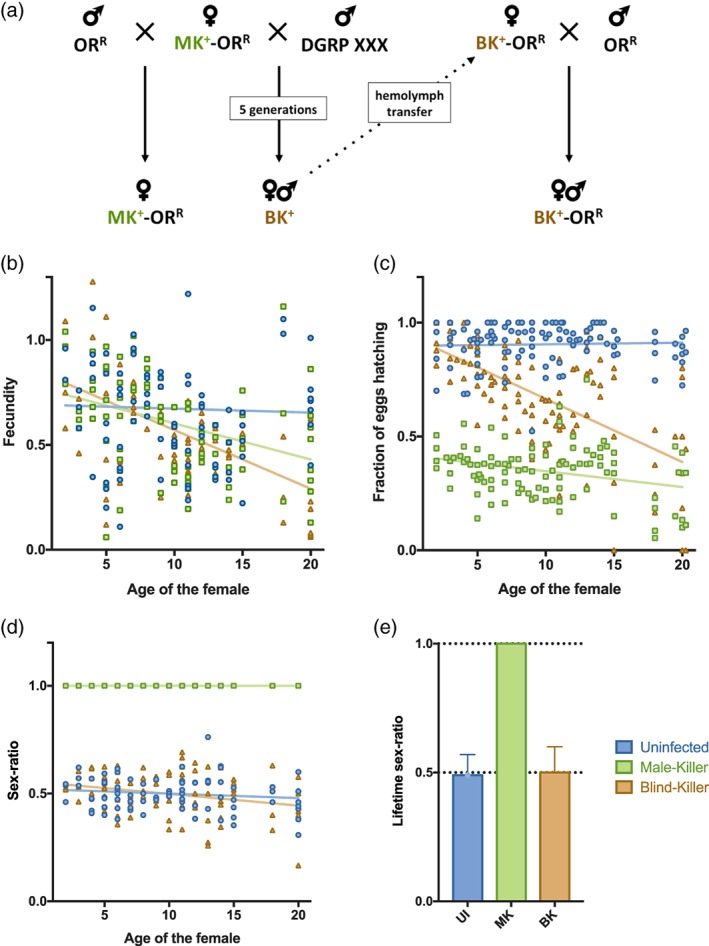
Discovery and phenotypic characterisation of the Blind‐Killer strain. (a) OR^R^ females infected by the MK *Spiroplasma* strain were backcrossed with males from a DGRP line. Several crosses reveal the appearance of males in the progeny. Hemolymph of infected females from these crosses was then injected back into OR^R^ females to eliminate any host background effect on male‐killing, leading to the establishment of a BK‐infected *Drosophila* stock. (b) Number of eggs laid by uninfected (UI—blue), MK‐infected (MK—green) and BK‐infected (BK—orange) flies of different age. Lines indicate linear regression fits (R^2^ for UI = 0.002; R^2^ for MK = 0.331; R^2^ for BK = 0.669). (c) Embryo viability from the same flies depending on their age when laying the eggs. Lines indicate linear regression fits (R^2^ for UI = 0.010; R^2^ for MK = 0.248; R^2^ for BK = 0.731). (d) Sex‐ratio of their offspring depending on their age when laying the eggs. Lines indicate linear regression fits (R^2^ for UI = 0.087; R^2^ for MK = 1; R^2^ for BK = 0.357). (e) Sex‐ratio of their offspring over their total lifespan (mean ± *SD*). Graphs display all data points acquired over four independent repetitions performed on a 3 years period

MK penetrance strongly relies on the parental genetic background (Kageyama, Anbutsu, Shimada, & Fukatsu, 2009). To investigate this effect, we crossed Oregon‐R (OR^R^) females infected with *S. poulsonii* Uganda‐1, a MK strain with full penetrance (hereafter named ‘MK strain’), with a hundred lines from the *Drosophila* Genetic Reference Panel collection (Mackay et al., 2012). Each subsequent generation was then backcrossed with the parental DGRP line for five generations to replace the OR^R^ background by the DGRP ones. Seven of these crosses gave a progeny that contained male individuals, thus showing a defective MK activity. This defective MK phenotype was however not observed systematically upon repeating the exact same crosses several times, suggesting that it appeared randomly because of yet unidentified factors (data not shown). We then injected hemolymph from these MK defective DGRP crosses back into the original OR^R^ background, where MK is fully penetrant. Interestingly, one newly infected OR^R^ background line retained a reduced MK penetrance, showing that this phenotype was independent of the host genetic background but was rather caused by a change in the *Spiroplasma* genotype (Figure 1a). The *Drosophila* OR^R^ females infected with this new *Spiroplasma* variant had a normal fecundity compared to that of the original *Spiroplasma* strain (Figure 1b), but the viability of the embryos decreased along with female aging, while embryo viability remained stable over aging of flies infected with the original strain (Figure 1c). The sex‐ratio of the offspring was however of 50% males and 50% females, as in uninfected flies (Figure 1d,e), suggesting that the new *Spiroplasma* variant kills embryos of aging females regardless of their sex. We hereafter name this phenotype ‘blind‐killing’ (BK) to oppose it to MK that selectively kills males.

**Figure 2:**
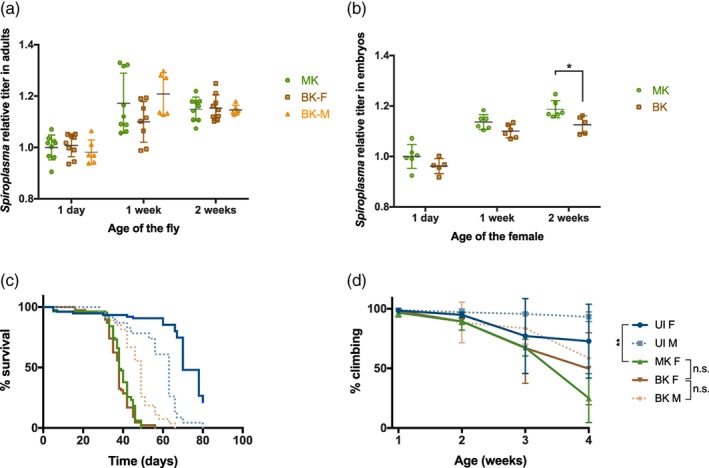
MK and BK *Spiroplasma* titre and effects on survival and neurodegeneration of *Drosophila*. (a) *Spiroplasma* titre in adult female infected by the MK *Spiroplasma* (MK‐F) and adults females and males infected by the BK *Spiroplasma* (BK‐F and BK‐M, respectively), as copy number of the *Spiroplasma* gene *dnaK* relative to host copy number of *rps17*. Two‐way ANOVA *P*‐value for MK/BK infection status: *P* = .4094. Three technical replicates from three independent experiments (two for BK‐M) are displayed as mean ± SD. (b) *Spiroplasma* titre in embryos collected from MK‐ and BK‐infected females of different age (time post‐eclosion is indicated), as copy number of the *Spiroplasma* gene *dnaK* relative to host copy number of *rps17*. Two‐way ANOVA *P*‐value for infection status: ***P = .001; Sidak's multiple comparisons test MK vs BK at 2 weeks: *P = .0207). Three technical replicates from three independent experiments (two for BK‐M) are displayed as mean ± SD. (c) Lifespan of virgin flies on standard diet. Female (F) or male (M) flies were non infected (UI), infected with the MK or the BK *Spiroplasma* strain. *n* = 284. Survival curves were analysed by log‐rank (Mantel‐Cox) test. *P*‐Value MK‐F vs BK‐F = 0.091; *P*‐value BK‐F vs BK‐M < .001. (d) Climbing activity of virgin flies. *n* = 20 flies per condition. Data were analysed using 2‐way ANOVA followed by post‐hoc Tukey HSD tests. BK,BK‐infected line; MK,MK‐infected line; **:*P*‐value < .01; n.s.: not significant (*P*‐value > .05); UI,uninfected line

In a previous report, a natural variant of *S. poulsonii* exhibiting weak male killing has been identified and the low MK phenotype was tied to a lower endosymbiont titre in the adult females. This suggested that a density threshold needs to be met for full MK penetrance (Anbutsu & Fukatsu, 2003). To determine whether this threshold hypothesis applies to the BK *Spiroplasma* strain we identified, we compared the bacterial titre in adults and eggs of MK‐ and BK‐*Spiroplasma* infected OR^R^ females. In adults (Figure 2a), no difference was observed between MK‐infected females titre and BK‐infected males and females. Embryos from BK‐infected females had a slightly lower titre than the MK‐infected ones (Figure 2b). The difference was however significant only for embryos deriving from mothers aged of 14 days, while the BK phenotype is already detectable in one‐week‐old mothers, suggesting that the BK phenotype and the endosymbiont titre are not correlated.

Adult flies infected by the original MK *Spiroplasma* strain have a strongly decreased lifespan compared to uninfected flies (Herren et al., 2014). We checked this trait in BK *Spiroplasma‐*infected flies, and found a similar lifespan for adult females infected by the BK and the MK *Spiroplasma* strains (Figure 2c). Males infected by the BK strain unexpectedly lived slightly longer than infected females (Figure 2c). Aging infected flies usually also display a phenotype suggestive of a neurodegeneration starting 3 weeks after emergence, including a loss of negative geotaxis and tremors (Herren et al., 2014). We monitored the loss of negative geotaxis in flies infected by the MK or the BK *Spiroplasma* strain, and found a similar intensity of decay at 3 and 4 weeks after emergence (Figure 2d). This suggests that the BK strain is not more deleterious than the MK strain for adults but rather differentially impacts *Drosophila* embryos. Therefore, we focused our analysis on early embryonic stages.

**Figure 3:**
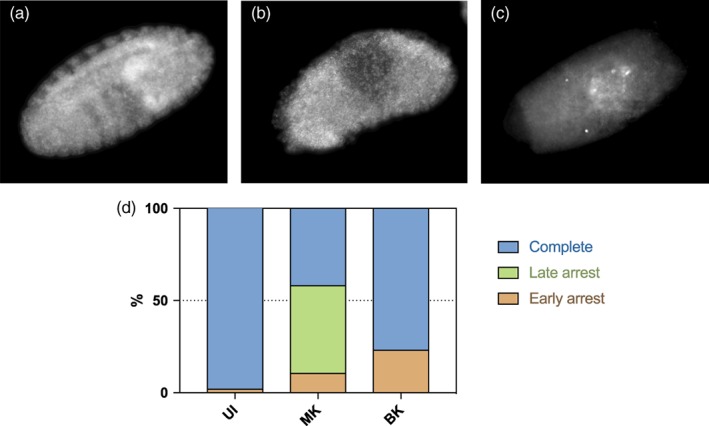
Scoring of embryos developmental arrest. Representative pictures of DAPI‐stained embryos aged 14–15 hr displaying (a) a normal phenotype (embryonic structures clearly identifiable), (b) a late death phenotype (embryonic structures roughly identifiable but misshapen) and (c) an early death phenotype (no clear structures identifiable). (d) Scoring of each arrest phenotype in UI,uninfected line (*n* = 53); MK,MK‐infected line (*n* = 124); BK,BK‐infected line (*n* = 91); (Chi‐square test *P*‐value < .001)

**Figure 4:**
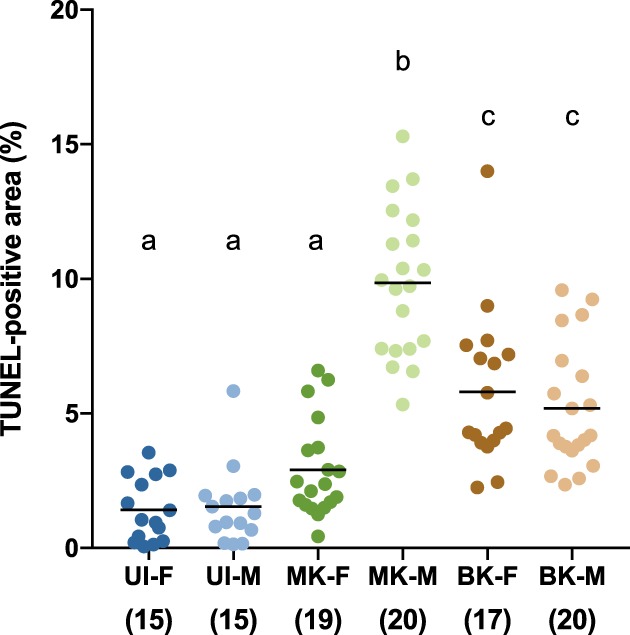
Quantification of apoptotic signal in stage 12 embryos. Numbers in brackets indicate the number of embryos examined. Each point represents one embryo, and black bars indicate means. Letters represent the clustering obtained by an ANOVA followed by a post‐hoc Tukey HSD test. BK, BK‐infected line; F, females; M, male; MK, MK‐infected line; UI, uninfected line

A 4',6‐diamidino‐2‐phenylindole (DAPI) staining of 14 to 15 hr old embryos showed that MK‐ and BK‐infected ones do not die at the same stages. 40% of MK‐infected embryos display a normal phenotype, while in 50% of them embryonic structures are identifiable but the global shape of the embryo looks askew, suggesting a death happening late in development (Figure 3). Last, 10% look like they did not properly start developing at all, as if their death happened during very early stages (Figure 3). BK‐infected embryos on the other hand display a normal phenotype for 75% and an early death phenotype for 25%. No late developmental arrest was observed for BK embryos. To further characterise embryonic development, we monitored apoptosis at stage 12 in MK‐ and BK‐infected embryos, as massive apoptosis, a hallmark of MK, takes place at this stage (Harumoto, Anbutsu, & Fukatsu, 2014). As expected, we detected a strong apoptotic signal in MK‐infected male embryos and not in females. In contrast, embryos infected by the BK strain displayed an intermediate level of apoptosis equivalent in both sexes (Figure 4). Furthermore, we did not observe any chromatin bridges in male embryos at stage 8–10 (data not shown), a feature associated with male killing. Altogether, our data suggest that BK‐infected embryo die at earlier developmental stages than MK‐infected ones, and that both male and female BK‐infected surviving embryos trigger apoptosis at similar levels lower than that observed in MK‐infected males. Last, two hallmarks of MK, male‐specific high levels of apoptosis and chromatin bridges are not found in embryos infected by the BK strain, suggesting that killing by this strain occurs through a distinct mechanism to that of MK.

### 2.2 Genome sequence of the BK strain

To better characterise this new variant, we sequenced the genome of the BK strain. Structural differences in the chromosome of the BK strain compared to the reference genome of the same strain published in 2015 (Paredes et al., 2015) could not be assessed with precision because of the fragmentation of the BK assembly in 23 contigs. However, 14 chromosomal Single‐Nucleotide Polymorphism (SNP) were identified, none of them affecting a gene predicted to be involved in the pathogenicity towards the host. We also noticed that the large plasmid of the MK strain (Masson et al., 2018) underwent a deletion of 8.8 kb in the BK strain (Figure S1). The deleted part contains coding sequences for the MK toxin Spaid (Harumoto & Lemaitre, 2018) and ParA1, a regulator of plasmid partition (Gerdes, Howard, & Szardenings, 2010), as well as seven small sequences (between 147 and 567 bp) coding for hypothetical proteins of unknown function. The deletion of *Spaid* is likely accountable for the loss of MK, confirming recent studies about the involvement of this toxin that were not yet validated by a null mutant for this gene (Harumoto & Lemaitre, 2018). The full‐length plasmid was still partially present in the BK *Spiroplasma* population in the sequencing data acquired in 2016. About 0.08 copies were detected per chromosomal copy, indicating that the sequenced bacterial population was constituted of a vast majority of recombinant cells, but also included some cells bearing the original (MK) version of the plasmid. A polymerase chain reaction (PCR) diagnostic done on *Spaid* in 2018, 2 years later, failed to detect it, suggesting that the BK population completely lost the full‐length plasmid within this time (Figure S2).

Furthermore, the rest of the plasmid appeared highly dynamic compared to the chromosome, with 23 SNPs over a 27.5 kb sequence although most of these were located in intergenic regions. This suggests that extrachromosomal DNA is likely to play a major role in the virulence of *S. poulsonii*, as it has been proposed for pathogenic species (Breton, Duret, Danet, Dubrana, & Renaudin, 2010; Davis et al., 2005; Saillard et al., 2008). Therefore, our genomic analysis of the BK strain explains the loss of MK in this strain and further highlights the importance of *Spaid* gene in this process.

### 2.3 Comparative transcriptomics of the MK and BK strains

Although the genome analysis points to the deletion of *Spaid* as the more probable cause of the loss of male‐targeting by the BK strain, it did not reveal any mutation that could explain the increased toxicity of the BK strain towards embryos of both sexes. We thus compared the transcriptome of MK‐ and BK‐*Spiroplasma* extracted from the hemolymph of adult flies. The transcriptome profile of BK *Spiroplasma* extracted from male flies was highly similar to that of extracted from females. Only one gene (SMSRO_SF024050) was significantly upregulated in males. This gene, which is restricted to the *Spiroplasma* genus, codes for an uncharacterised protein, and homology‐based investigations failed at identifying its putative function. We thus assumed that *Spiroplasma* BK does not behave differentially in the hemolymph of male and female flies, and used only the female dataset for further analyses.

**Figure 5:**
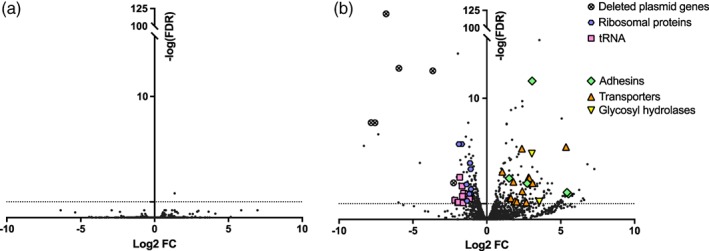
Volcano plot of differential gene expression of (a) *S. poulsonii* BK in male flies versus *S. poulsonii* BK in female flies and (b) *S. poulsonii* BK in female flies versus *S. poulsonii* MK in female flies (positive FC indicate an up‐regulation in BK from males compared to BK from females and in BK compared to MK respectively). Each point represents the average value of one transcript in three biological replicates. The dotted horizontal line indicates the significance threshold (FDR < 0.05)

The comparison between MK‐ and BK‐*Spiroplasma* extracted from female flies yielded 233 differentially expressed genes, of which 50 (21%) could be annotated automatically and 79 (34%) could be annotated manually (Figure 5). The remaining 104 (45%) sequences had no similarity to the sequences in the nr database when Psi‐BLAST was used as a search engine. The low number of annotated differentially expressed genes makes it difficult to obtain reliable information from global analyses such as GO enrichment or KEGG pathway analysis, leading us to analyse the dataset manually. The complete analysis can be found in Table S1.

We first examined the expression profile of genes affected by SNPs and by the partial plasmid deletion in the BK genome. Among the 16 chromosomal SNPs, 15 were located in or close to genes that are either undetected in the RNAseq or which expression is not differential between MK and BK strains. The remaining SNP affected the terminase‐coding gene SMSRO_SF011260, which had a low and variable basal expression unlikely to significantly affect *Spiroplasma* virulence.

As expected, *Spaid*, *parA1* and other hypothetical protein coding genes located on the plasmid fragment missing in the BK genome were the most downregulated genes in the BK transcriptome, confirming the deletion. Intriguingly, *parA2*, a second plasmid‐located copy of the *par* family, was also downregulated in BK strain.

We also found 13 genes encoding for various transporters upregulated in BK strain, including amino acids, ascorbic acid, phosphate, chromate and two glucose transporters. Genes encoding for an endo‐β‐N‐acetylglucosaminidase and a glycosylhydrolase, which can process free oligosaccharides and release monosaccharides (Davies & Henrissat, 1995; Suzuki et al., 2002), were also upregulated in the BK strain.

A strikingly high number of genes related to translation were downregulated in BK strain, including rplS, T and X (50S ribosomal proteins), rpsG, O, P, R, S and T (30S ribosomal proteins), eleven sequences coding for transfer RNAs (tRNA) including that carrying methionine (Met), and three genes coding for tRNA modification enzymes (*trmK*, *tadA* and *gatB*).

We then explored whether virulence genes previously annotated in *S. poulsonii* genome were differentially expressed between the BK strain and the MK strain. Five genes coding for putative virulence‐related adhesins (p123, p54 and p18) were found to be upregulated in the BK strain transcriptome. However, among the 15 virulence genes beside adhesins that were described in the latest genome annotation (Masson et al., 2018), only three had a differential expression level between the strains, including the gene coding for the membrane lectin Spiralin B (Killiny, Castroviejo, & Saillard, 2005), for the glycerol‐3‐phosphate oxidase GlpO that produces radical oxygen species (Vilei & Frey, 2001) and the protective toxin RIP2 (Hamilton et al., 2016). This notwithstanding, all of them were downregulated in BK strain compared to MK.

In conclusion, the trancriptomics of the BK strain did not reveal any overt toxicity mechanism that would explain the embryonic mortality. We however found a stronger line of evidence in favour of a metabolic shift of the bacteria that would affect the embryonic development regardless of its sex, rather than a direct pathogenic effect mediated by virulence factors.

### 2.4 Protection against parasitoid wasps

**Figure 6:**
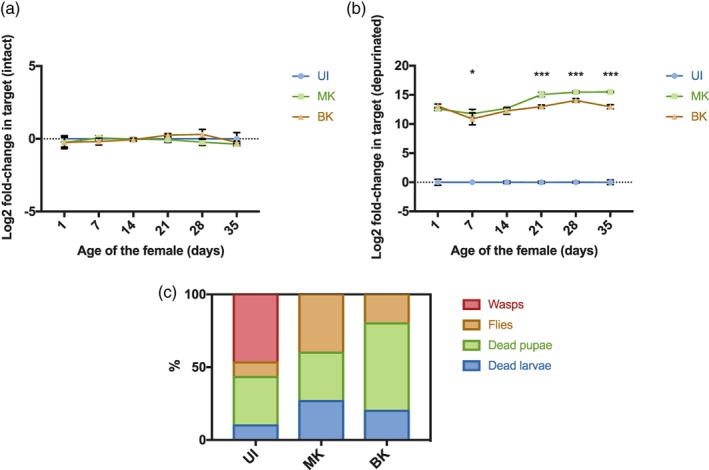
The BK strain displays RIP activity and confers protection against parasitoid wasps. RIP activity in flies uninfected (UI), or infected with the MK or BK strain of *S. poulsonii* monitored by the respective abundance of (a) intact and (b) depurinated *Drosophila* rRNA template. Two‐way ANOVA, *P*‐value for factor ‘infection’ = .0074. Stars indicate significance upon multiple *t*‐testing of MK vs BK with Holm‐Sidak correction (*P*‐value* < .05; *P*‐value*** < .001). (c) Quantification of *D. melanogaster* dead larvae and pupae, emerging fly adults, and wasp adults upon *Leptopilina boulardii* infestation (*P*‐value*** < .0001; Chi‐square = 60.63; df = 6). One representative experiment is shown out of five

*Spiroplasma*‐mediated protection against parasitoid wasps and nematodes involves RIP toxins that are encoded by five chromosomal genes (Hamilton et al., 2016), of which only *RIP1* and *RIP2* are significantly expressed, *RIP2* being the most expressed (Garcia‐Arraez, Masson, Escobar, & Lemaitre, 2019). The down‐regulation of *RIP2* in the BK strain thus raised the question of the ability of the BK strain to protect the fly against natural enemies. We monitored RIP activity over the lifespan of adult females infected with the MK or the BK strain and observed a similar level for flies up to 2 weeks old, but a lower RIP activity in BK‐infected flies aged 3 weeks and more (Figure 6a,b). We observed a high mortality in pupae developing from larvae infected by the BK strain and challenged with the parasitoid wasp *Leptopilina boulardii*, but the surviving individuals were overall resistant as no wasp emerged from most biological replicates (Figure 6c). We however observed two wasps emerging from BK‐infected larvae on a total of 150 larvae examined while this never happened with MK‐infected larvae. These two escaper wasps suggest that the protection conferred by the BK strain, although very efficient, might be weaker than that of the MK strain.

## 3 DISCUSSION

We isolated a spontaneous variant of *S. poulsonii* Uganda‐1 that does not cause MK in its host *D. melanogaster* but rather kills unselectively a subset of both male and female progeny, a phenotype we named BK.

A first key feature of the BK strain is the loss of MK, that is, the killing of male‐embryos specifically. Sequencing the BK strain genome revealed a deletion of a fragment of the natural plasmid encompassing the gene encoding for the MK toxin Spaid (Harumoto & Lemaitre, 2018), *parA1* and seven yet uncharacterised other genes. The plasmid deletion happened between repetitive sequences, likely by homologous recombination, a rare phenomenon in *Spiroplasma* because of the pseudogenisation of the crucial recombination gene *recA* and the loss of other genes of the rec family (Carle et al., 2010; Davis et al., 2005; Saillard et al., 2008). The sequence coding for the replication protein pE, which must be borne on the plasmid to ensure its replication (Breton, Duret, Arricau‐Bouvery, Béven, & Renaudin, 2008), is intact and not differentially expressed, suggesting that the plasmid replication is functional in the BK strain. However, the deletion of *parA1* and down‐regulation of *parA2* suggests that the partition of the plasmid could be less efficient and potentially lead to its loss.

With the deleted plasmid fragment being undetectable by PCR, the BK strain can be considered as a natural knock‐out of *Spaid*, thus confirming the function of this gene as a necessary MK factor. Although it recently became cultivable in vitro (Massonet al., 2018), *S. poulsonii* is not genetically tractable yet, making such natural mutants precious tools for further studies on the MK mechanism.

The absence of Spaid coding gene in the BK genome can most likely be accounted for the inability of the bacteria to selectively kill male embryos (although we cannot exclude that one or several of the other deleted genes also participates to MK) but the death of a subset of infected embryos of both sexes is surprising and remains mechanistically unclear. The MK strain kills on average 65% of the progeny (100% of males and around 25% of the female embryos) (Figure 1 and (Garcia‐Arraez et al., 2019)). The BK strain kills both male and female embryos with increased lethality when the mother ages: from about 20% for few days‐old mothers, to 40% for mothers over 1 week old. This indicates that the BK strain is more deleterious than the MK strain for female embryos, although the bacterial titre is identical or lower. The deleterious effect does not affect adult flies, as lifespan and neurodegenerative phenotypes were similar between MK‐ and BK‐infected flies (Figure 2). RNAseq analysis showed that virulence factors were either not differentially expressed or downregulated in BK, with the exception of adhesin coding genes. Adhesin‐like proteins have been shown to be necessary for pathogenic *Spiroplasma* species to adhere to and invade host cells (Béven et al., 2012; Breton et al., 2010; Hou et al., 2017; Zha, Yang, Wang, Yang, & Yu, 2018), these could thus be responsible for the BK strain increased pathogenicity. Future studies are required to address the role of these proteins in endosymbiotic *Spiroplasma* and determine whether they are involved in the BK phenotype.

An alternative hypothesis would be that the BK toxicity rather comes from a shift in the bacterium metabolic activity that kills the embryo in a sex‐independent fashion. The RNAseq revealed two BK features related to metabolism: an up‐regulation of transporters and glycosidases, and a down‐regulation of translation‐related genes. tRNAs up‐regulation is a mechanism that allow bacteria in co‐cultures to grow better in a medium where there is competition for nutrients (Tognon, Köhler, Luscher, & van Delden, 2019). It is also a hallmark of cancer cells, where increased tRNAs can increase the translation of mRNA that bear their cognate codons (Goodarzi et al., 2016). tRNA regulation could thus be a way for *Spiroplasma* to modulate protein production and, in the case of BK, to decrease the activity of the whole translation machinery through the down‐regulation of tRNA‐Met. A decreased metabolic activity, which along with the up‐regulation of transporters and glycosyl hydrolases, could indicate that BK relies more on nutrient uptake for its host than MK *Spiroplasma* to sustain its proliferation. Since the BK titre is not significantly different from MK in adults and eggs laid by young mothers (Figure 2), we assume that the metabolic shift of BK has little consequences on adult hosts that have unlimited access to food. In embryos, however, where resources are limited, the increased uptake capacity of the BK strain would prop up the competition for available nutrients, causing detrimental effects on *Drosophila* embryonic development and viability. The eggs viability would thus decrease along with *Spiroplasma* titre, which is consistent with our phenotypic observations (Figures 1c and 2b).

Remarkably, this is the second strain described in the literature within a 2‐years period as a natural variant that lost MK ability (Harumoto & Lemaitre, 2018). In lab stocks, MK is used as a proxy to assess the infection status. If males emerge from the progeny of infected flies, the whole progeny is considered as uninfected and discarded, while potentially being well‐infected by a natural non‐male‐killer variant. The frequency of such variants is thus likely to be underestimated in lab conditions. This also suggests that MK could be quite unstable in the absence of positive selective pressure, notably because of the plasmid location of *Spaid* (while other phenotypes such as protection against natural enemies are mediated by chromosomal, thus more stable genes).

The prevalence of endosymbionts capable of MK is highly variable between species, ranging from 1% for *S. poulsonii* in *Drosophila willistoni* (Williamson & Poulson, 1979) up to 99% for *Wolbachia* infecting some populations of the butterfly *Hypolimnas bolina* (Dyson & Hurst, 2004), and also within populations of the same species (Kageyama et al., 2009). Most associations with MK endosymbionts are recent and thus likely transient, indicating that fixation of the symbiont in the host population leads in most cases to the extinction of the host species because of the paucity of males (Dyer & Jaenike, 2004; Hatcher, Taneyhill, Dunn, & Tofts, 1999). Some examples of long‐lasting associations have been discovered but their evolutionary stability despite of MK remains unexplained (Dyer & Jaenike, 2004). A current hypothesis is that hosts undergo pressure to evolve resistance mechanisms against MK, although experimental evidence of the emergence of such resistances is scarce (Dyson & Hurst, 2004; Hayashi, Nomura, & Kageyama, 2018; Jiggins, Hurst, & Yang, 2002). Our study suggests that an extremely stable environment (constant temperature, no competition for food, no predators, forced inbreeding) favours the apparition of non‐MK variants, which could be a step towards fixation of the endosymbiont. The ability to protect its host against parasitoid wasps, a key mechanism of endosymbiont spread along with MK (Jaenike, Unckless, Cockburn, Boelio, & Perlman, 2010), and an efficient vertical transmission would then be the major selective pressures maintaining these variants. The persistence of originally MK endosymbionts could thus rely either on the emergence of a host resistance to MK, or on a symbiont adaptation to lose (or reduce) its MK ability as long as other positive selective pressures such as protection against natural enemies are still operating.

## 4 EXPERIMENTAL PROCEDURES

### 4.1 Fly and *Spiroplasma* stocks

Flies were kept at 25°C on standard cornmeal medium (35.28 g of cornmeal, 35.28 g of inactivated yeast, 3.72 g of agar, 36 ml of fruits juice, 2.9 ml of propionic acid and 15.9 ml of Moldex for 600 ml of medium). We used a wild‐type Oregon‐R (OR^R^) fly stock cured from *Wolbachia* and infected by the *Spiroplasma poulsonii* MSRO strain Uganda‐1 (Herren & Lemaitre, 2011) years prior to this work. DGRP infection was performed by injecting them with 9 nl of undiluted hemolymph extracted from infected females using a Nanoject II (Drummond) as previously described (Herren & Lemaitre, 2011). The DGRP line from which we isolated the BK strain was #25191.

### 4.2 Phenotypic characterisation

Flies uninfected, MK‐ and BK‐infected were collected at emergence from pupae and mated 3 days later with OR^R^ uninfected males. Fecundity was measured by transferring flies in new tubes twice a day for 3 weeks. The number of eggs in each tube was counted and normalised by the number of females and number of hours of laying. The hatching rate was determined by counting the number of unhatched eggs in the same tubes after 48 hr of incubation at 25°C. The number and sex‐ratio of the offspring was determined after 12 more days at 25°C. These experiments have been performed twice in 2016, once in 2018 and once in 2019 with no significant difference between replicates. Titre measurements have been performed by qPCR as previously described (Herren & Lemaitre, 2011) using the ΔΔCT method to quantify the abundance of *Spiroplasma dnaK* copy number relative to host *rps17* copy number. Titre measurements have been performed three independent times in adults except BK‐M (two times), and two times in embryos. Survivals and negative geotaxis assays (climbing assays) as well as DAPI and Terminal deoxynucleotidyl‐transferase dUTP nick end labeling staining and quantification have been performed as described, along with msl‐1 staining used to sex the embryos (Harumoto et al., 2014; Herren et al., 2014; Herren & Lemaitre, 2011). These experiments were repeated three independent times, except for DAPI staining that has been performed once. Wasp challenges have been performed using the progeny of 1‐week‐old mothers as previously described (Paredes et al., 2016) and were repeated five independent times. RIP assays were performed by reverse transcription quantitative PCR (RT‐qPCR) as previously described (Hamilton et al., 2016) and were repeated three independent times.

### 4.3 Plasmid detection by PCR

DNA from pools of 5 flies was extracted as previously described (Herren & Lemaitre, 2011). PCRs were carried out on total DNA using GoTaq G2 with the following cycling protocol: 1 min at 95°C, 35 cycles of 30 s at 95°C, 30 s at 60 or 65°C, 2 min at 72°C and a final step of 5 min at 72°C. Primers for *dnaA* are published (Herren & Lemaitre, 2011) and were used at 60°C. Primers for *Spaid* (F‐ACCAACAACAATTGCCGCT; R‐GCTAAGGCAAGCGCGGTA) and *soj* (F‐TGAACGCACTCGCAAAGC; R‐GACACTGCTTGCTCCGGT) were used at 65°C.

### 4.4 Genome sequencing, assembly and annotation

*S. poulsonii* DNA was extracted from fly hemolymph as previously described (Masson et al., 2018). Processing of the samples was performed in the University of Lausanne Genomic Technologies Facility. The DNA was sheared in a Covaris g‐TUBE (Covaris, Woburn, MA, USA) to obtain 20 kb fragments. The DNA size distribution was checked on a Fragment Analyser (Advanced Analytical Technologies, Ames, IA, USA). 2.1 μg of the sheared DNA was used to prepare a SMRTbell library with the PacBio SMRTbell Template Prep Kit 1 (Pacific Biosciences, Menlo Park, CA, USA) according to the manufacturer's recommendations. The resulting library was size selected on a BluePippin system (Sage Science, Inc. Beverly, MA, USA) for molecules larger than 6 kb. The recovered library was sequenced on one SMRT cell with P6/C4 chemistry and MagBeads on a PacBio RSII system (Pacific Biosciences, Menlo Park, CA, USA) at 240 min movie length.Assembly was performed with HGAP version 2 from the PacBiosmrtpipe (v2.3.0). Contigs were ordered according to the reference genome Spiroplasma poulsonii MSRO (Accession: NZ_JTLV00000000.2) using Abacas tool version 1.3.1 (Assefa, Keane, Otto, Newbold, & Berriman, 2009). Plasmid contig was circularised using Minimap2 tool (Li, 2018). Genome annotation was performed with Prokka v 1.12 (Seemann, 2014) using parameters: addgenes, genus Spiroplasma, species poulsonii, gcode 4, evalue 1e‐06, use genus, raw product, proteins MSROv2_proteins.fa, increment 10, rfam.

### 4.5 RNA‐sequencing

Total RNA was extracted by the TRIzol method, following the manufacturer's instructions, from the hemolymph of 30 one‐week‐old infected flies pelleted by 30 min centrifugation at 16,000*g*. Three independent replicates were prepared for each condition. RNA quality was assessed on a Fragment Analyser (Advanced Analytical Technologies, Inc., Ankeny, IA, USA). RNA‐seq libraries were prepared using 25 ng of total RNA and the Illumina TruSeq Stranded mRNA reagents (Illumina; San Diego, CA, USA) without the polyA selection step. Sequencing using Illumina HiSeq 2500 system was performed at the University of Lausanne Genomic Technologies Facility. Quality of the reads was checked using FastQC v 0.11.5 (available from: http://www.bioinformatics.babraham.ac.uk/projects/fastqc/), and low‐quality reads were discarded prior to trimming the adapters and low quality regions from the remaining reads using Cutadapt v 1.9.1 (Martin, 2011). Then, reads were mapped to *Spiroplasma poulsonii* MSRO reference genome (version NZ_JTLV00000000.2) using STAR v. 2.5.4b (Dobin et al., 2013). Read counts per gene were determined using the function htseq‐count from HTseq v. 0.9.1(Anders, Pyl, & Huber, 2015). Reads aligning uniquely to *S. poulsonii* transcripts were used to calculate differential gene expression using EdgeR package v 3.22.5 (Robinson, McCarthy, & Smyth, 2010) for R v 3.5.1. Significance was determined using EdgeR exact test for the negative binomial distribution, corrected with a False Discovery Rate (FDR) at *P* < .05.

### 4.6 Statistical analyses

Statistical analyses and graphs have been made using GraphPad Prism version 7.0a (GraphPad Software Inc., San Diego, CA). Data for fecundity, hatching rate, titre, sex‐ratio and climbing activity were analysed by analysis of variance (ANOVA) followed by post‐hoc testing if at least one of the factors was significant in the ANOVA (detailed in figure legends). Survivals were analysed using Log‐rank (Mantel‐Cox) test. Wasp challenge data were analysed using Chi‐square test.

## CONFLICT OF INTEREST

The authors declare no competing interests.

## AUTHOR CONTRIBUTIONS

F.M., S.R., J.C.P. and B.L. designed the project. G.G.A. and J.C.P. isolated the BK strain. F.M. and F.S. performed the experiments. S.C.C. performed the genome assembly and annotation and transcriptome assembly. A.V. performed the transcriptome assembly and analysis. The first draft was written by F.M. and edited by S.R., F.M. and B.L. All authors read and approved the final manuscript.

## Supporting information


**Figure S1.** Plasmid sequence comparison between MK and BK *Spiroplasma* strains. Blue (outer ring) and purple (inner ring) indicate coding sequences for hypothetical proteins in the MK and BK sequence respectively. Annotated genes are displayed in other colours with their name. Orange and red marks in the inner grid indicate repetitive sequences. The deleted fragment in the BK sequence including *Spaid* and *parA* is indicated by a plain grey line.

**Figure S2.** PCR detection of the natural plasmid of *Spiroplasma poulsonii*. Tracks 1–2: PCR on the chromosomal locus of *dnaA* (control); tracks 3–4: PCR on the plasmid fragment deleted in the BK strain; tracks 5–6: PCR on a plasmid region intact in the BK strain.

**Table S1.** Detailed EdgeR analysis of *Spiroplasma poulsonii* transcriptome MK versus BK.

## Data Availability

BK‐strain genome data have been submitted as part of the BioProject PRJNA256019 with the accession number SSBE00000000. RNAseq data have been submitted to GEO under the accession number GSE129674.
