# Supplementary figures and images for: Blind killing of both male and female Drosophila embryos by a natural variant of the endosymbiotic bacterium Spiroplasma poulsonii
Source: Cell Microbiol. 2020 Jan 23;22(5):e13156. doi: 10.1111/cmi.13156 (PMC7187355; doi:10.1111/cmi.13156)

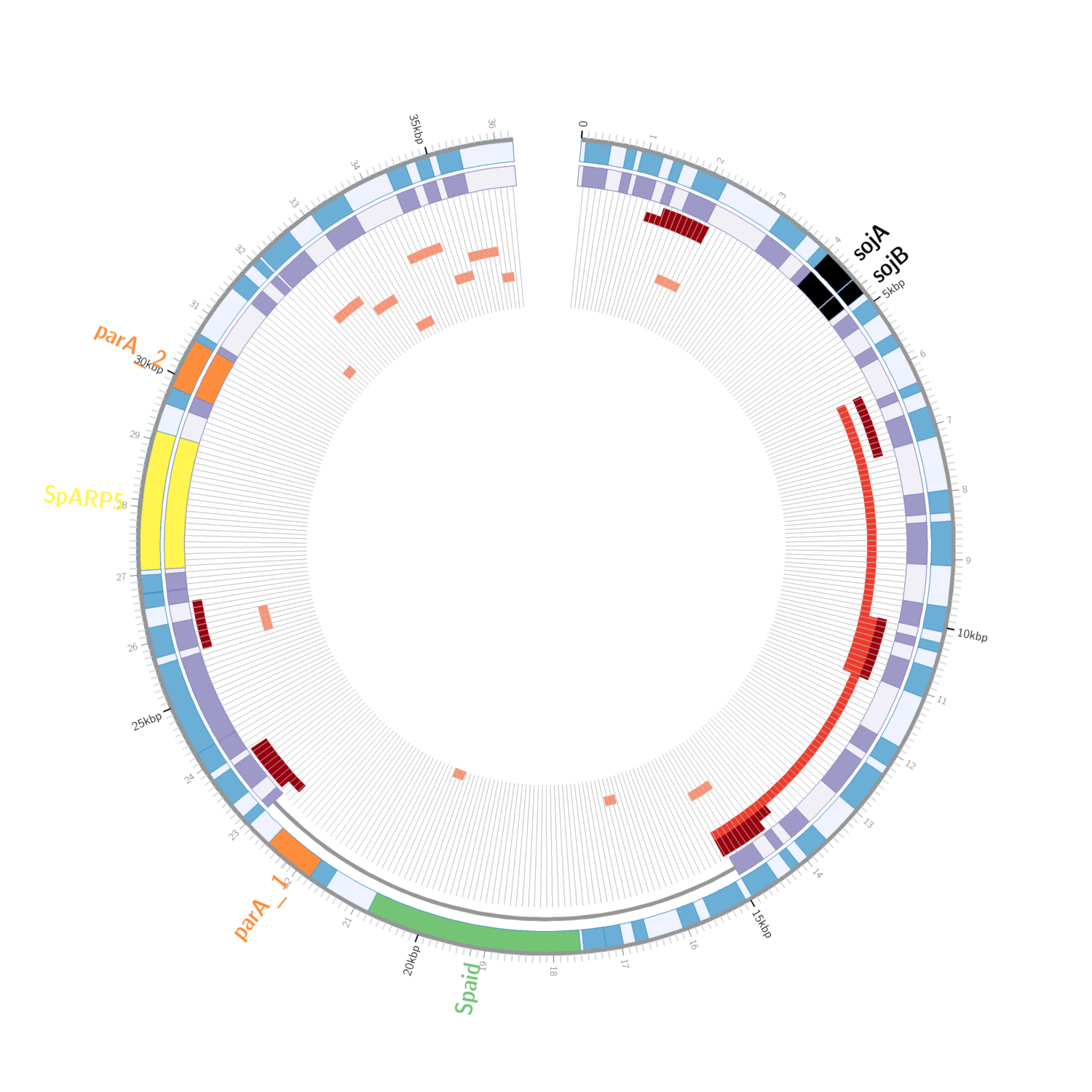

Supplement: Supplementary file 1 — Figure S1. Plasmid sequence comparison between MK and BK Spiroplasma strains. Blue (outer ring) and purple (inner ring) indicate coding sequences for hypothetical proteins in the MK and BK sequence respectively. Annotated genes are displayed in other colours with their name. Orange and red marks in the inner grid indicate repetitive sequences. The deleted fragment in the BK sequence including Spaid and parA is indicated by a plain grey line. [file CMI-22-e13156-s001.tif]

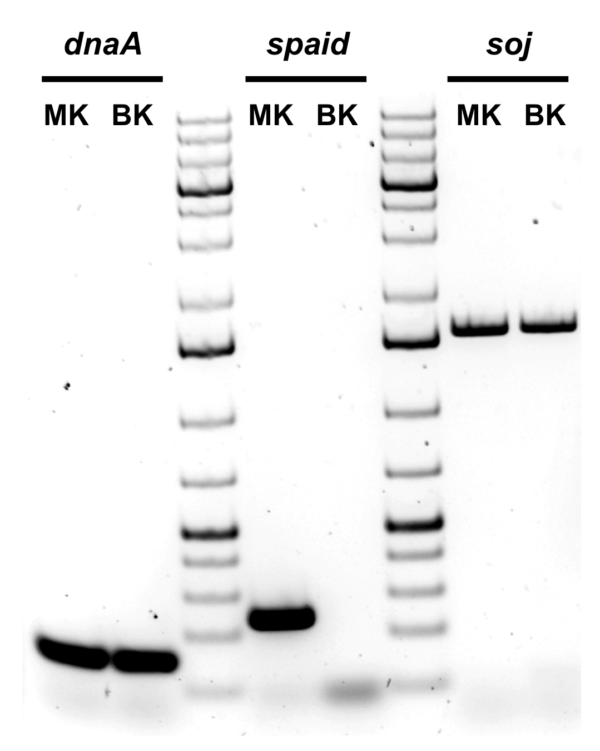

Supplement: Supplementary file 2 — Figure S2. PCR detection of the natural plasmid of Spiroplasma poulsonii. Tracks 1–2: PCR on the chromosomal locus of dnaA (control); tracks 3–4: PCR on the plasmid fragment deleted in the BK strain; tracks 5–6: PCR on a plasmid region intact in the BK strain. [file CMI-22-e13156-s002.tiff]
